# 驱动基因阳性非小细胞肺癌脑膜转移临床诊疗中国专家共识（2026版）

**DOI:** 10.3779/j.issn.1009-3419.2025.102.39

**Published:** 2026-01-20

**Authors:** 中国医药教育协会肺癌医学教育专业委员会 北京医学奖励基金会肺癌医学青年专家委员会转移性肺癌协作组

**Keywords:** 肺肿瘤, 脑膜转移, 驱动基因, 靶向治疗, 临床诊疗指南, Lung neoplasms, Leptomeningeal metastasis, Actionable gene alterations, Targeted therapy, Clinical practice guidelines

## Abstract

非小细胞肺癌（non-small cell lung cancer, NSCLC）脑膜转移（leptomeningeal metastasis, LM）的预后极差，然而在驱动基因阳性患者中，高中枢神经系统（central nervous system, CNS）渗透性靶向药物的出现，正显著重塑治疗格局。现有指南多倾向于保守策略，已难以满足精准医学的需求。为应对此挑战，北京医学奖励基金会肺癌医学青年专家委员会转移性肺癌协作组组织肿瘤内科、影像学、病理学、放疗及神经外科等多学科专家，基于近10年的循证医学证据及临床实践经验，制定了《驱动基因阳性非小细胞肺癌脑膜转移临床诊疗中国专家共识（2026版）》。本共识强调，LM诊断应综合临床症状、磁共振成像（magnetic resonance imaging, MRI）及脑脊液（cerebrospinal fluid, CSF）细胞学结果进行判断，并突出CSF分子活检在明确驱动基因状态及疗效评估中的关键作用。在治疗方面，本共识倡导根本性范式转变：由多学科诊疗（multidisciplinary team, MDT）团队主导治疗，以高CNS渗透性靶向治疗作为一线干预措施，局部治疗（如鞘内注射及放疗）则定位为辅助手段；共识针对高CNS渗透性酪氨酸激酶抑制剂（tyrosine kinase inhibitors, TKIs）的选择、鞘内化疗的应用、放疗时机及外科姑息治疗等关键环节，提供了一系列专家推荐，旨在为中国NSCLC LM患者确立基于分子分型和MDT协作的积极个体化治疗规范，从而提升患者生存水平。

## Abstract (en)

The prognosis for non-small cell lung cancer (NSCLC) with leptomeningeal metastasis (LM) is extremely poor. However, in oncogene addicted patients, the emergence of targeted therapies with high central nervous system (CNS) penetration is significantly reshaping the treatment landscape. Existing guidelines, which often favor conservative strategies, are no longer sufficient to meet the demands of precision medicine. To address this challenge, the Metastasis Lung Cancer Collaboration Group, Youth Specialists Committee of Beijing Medical Reward Foundation convened multidisciplinary experts from Medical Oncology, Radiology, Pathology, Radiotherapy, and Neurosurgery. Based on evidence-based medicine from the past decade and clinical practice experience, they have developed the Chinese expert consensus on clinical management of oncogene addicted NSCLC with leptomeningeal metastasis (2026 edition). This consensus emphasizes that the diagnosis of LM should be based on a comprehensive assessment of clinical symptoms, magnetic resonance imaging (MRI), and cerebrospinal fluid (CSF) cytology. It also highlights the critical role of CSF molecular liquid biopsy in clarifying driver gene status and assessing treatment efficacy. In terms of treatment, this consensus advocates for a fundamental paradigm shift: therapy should be led by a multidisciplinary team (MDT), with high-CNS-penetration targeted therapies as the first-line intervention, while local treatments (such as intrathecal injection and radiotherapy) are positioned as supplementary measures. The consensus provides a series of expert recommendations on key aspects, including the selection of high-CNS-penetration tyrosine kinase inhibitors (TKIs), the application of intrathecal chemotherapy, the timing of radiotherapy, and palliative surgery. It aims to establish proactive, individualized treatment standards for patients with NSCLC LM in China, based on molecular subtyping and MDT collaboration, thereby improving patient survival outcomes.

自1869年Eberth首次报道肿瘤柔脑膜转移（leptomeningeal metastasis, LM）以来^[1]^，实体瘤LM因其进展迅速并缺乏有效的干预手段，且发生机制尚未完全阐明，一直被视为预后极差的实体瘤并发症。LM指肿瘤细胞突破血脑屏障（blood-brain barrier, BBB），通过脑脊液（cerebrospinal fluid, CSF）在软脑膜及蛛网膜下腔的弥漫性播散^[2,3]^或直接浸润柔脑膜表面^[4]^。需注意的是，LM并不包括经血行转移累及硬脑膜（pachymeninx）的病变，因后者不涉及CSF途径的肿瘤播散。此外，与脑实质转移不同，发生LM的肿瘤细胞具备依附性和悬浮生长的双重能力^[5]^，表现为两种表型：一种是肿瘤细胞附着于柔脑膜，形成实体结节；另一种是漂浮在CSF中无锚生长^[3,5]^。肿瘤细胞在整个中枢神经系统（central nervous system, CNS）内广泛播散，常常导致进行性神经功能障碍、亚急性脑积水等严重症状，故LM也被称为柔脑膜癌病变（leptomeningeal carcinomatosis）、癌性脑膜炎（neoplastic/carcinomatous meningitis）^[6-8]^。

虽然缺少大规模的流行病学调查，但小样本回顾性研究^[3,9]^显示肺癌是最常发生LM的实体瘤类型之一（3%-9.4%），尸检报告提示发生率可能高达25%，侵袭性较强、存在驱动基因变异的非小细胞肺癌（non-small cell lung cancer, NSCLC）患者则更容易发生LM。回顾性研究^[10,11]^发现，表皮生长因子受体（epidermal growth factor receptor, EGFR）突变NSCLC患者的LM发生率显著高于野生型（9.4% vs 1.7%）；由于诊断技术的局限性，影像学和CSF细胞学敏感性不足，LM实际发生率可能被低估^[12]^。回顾性研究^[13,14]^发现，肺癌患者从原发癌确诊到LM发生的中位时间通常为8-15个月；大多数患者确诊时已出现头痛、恶心、肢体无力、视物模糊、耳鸣等复杂多变的CNS症状，仅少数患者表现为无症状脑膜种植^[15]^。

LM起病隐匿但进展迅速，未经治疗的患者中位生存期仅2-4个月^[16]^。其预后差主要是因为肿瘤细胞在CSF乏氧和生长因子缺乏的恶劣环境下仍能通过代谢适应性存活，即便鞘内化疗也难以完全控制^[5,17,18]^。因此，既往临床干预多以保守和对症治疗为主，目标在于延缓神经功能恶化，并在维持生活质量的前提下延长生存期^[2]^。

近年来，CSF细胞学和液体活检技术的快速发展显著提高了LM的诊断敏感性，为早期诊断提供了可能。同时，针对驱动基因阳性NSCLC患者，靶向治疗显著改善了生存期，LM的治疗格局也随之改变^[19]^。一项回顾性研究^[20]^证实，接受靶向治疗、化疗并联合抗血管生成方案治疗的肺癌LM患者死亡风险显著降低了76%；提示以EGFR和间变性淋巴瘤激酶（anaplastic lymphoma kinase, ALK）抑制剂为代表的靶向药物能有效渗透CSF^[21,22]^，提升颅内治疗效果，成为延长驱动基因阳性LM患者无进展生存期（progression-free survival, PFS）的关键^[23,24]^。此外，鞘内注射也开始逐渐从传统的化疗药物（甲氨蝶呤、噻替哌、拓扑替康）转为培美曲塞^[25]^，这些新疗法均被证实提升了LM患者的总生存期（overall survival, OS）。

美国国立综合癌症网络（National Comprehensive Cancer Network, NCCN）指南曾提出将实体瘤LM患者分为低危和高危两类。低危人群通常表现为较好的体能状况、无重大神经功能缺损、较少的伴随疾病等，对放化疗耐受性好，建议进行强化全身治疗；相较之下，高危人群则体能状态欠佳，常伴有显著的神经功能障碍，伴发多种或较严重系统性疾病，多脏器功能受损，预后不良且预期生存期有限，对系统治疗的耐受性较低，推荐以支持治疗为主^[26]^。显然这种分类方法已经不适应目前精准治疗的发展，特别是对于驱动基因阳性的LM人群，会错失更优的生存获益。因此，协作组认为对于驱动基因阳性NSCLC患者的LM，治疗不应再拘泥于“保守”；在有多学科诊疗（multidisciplinary team, MDT）团队充分支持的基础上，可以采取更为积极的治疗手段；故对驱动基因阳性NSCLC患者制定新的LM共识是有必要的。因此，我们亟需基于分子分型和MDT协作，制定新的诊疗共识，以实现驱动基因阳性NSCLC LM更精准、有效的个体化治疗。

在这一背景下，北京医学奖励基金会肺癌医学青年专家委员会转移性肺癌协作组召集了肿瘤内科、影像、病理、检验、放疗、神经外科领域的专家学者，共同制定了这部《驱动基因阳性非小细胞肺癌脑膜转移临床诊疗中国专家共识（2026版）》。本共识旨在推动基于分子分型和MDT的优化方案，以应对LM的双表型特性，并加速其向临床实践转化，从而提升LM患者的生存基准，并为未来的临床研究提供参考，助力提升我国肺癌的综合诊治水平。

## 1 方法

编写组主要收集并评估了近10年（截止至2025年10月）驱动基因阳性NSCLC LM的诊疗循证依据，考虑到此类研究普遍样本量不足且缺乏III期临床研究，更多是探索性的I、II期研究。因此对于证据的分级，编写组在GRADE标准^[27]^的基础上进行了更新，以适应LM循证的特点（表1）。在此基础上，编写组重点针对CSF细胞学及基因诊断价值、系统治疗与局部治疗的时机和顺序、鞘内注射药物选择、放疗方式等临床争议问题进行了论述并形成共识。本共识不仅采纳了新的循证依据，也融入了编写组专家的临床经验，兼顾可操作性和药物、诊断设备的可及性。需要指出的是，本共识仅针对驱动基因阳性NSCLC LM诊治，原发肿瘤相关的分子病理诊断请参考相应指南。若同时合并脑实质转移，建议参考《中国驱动基因阳性非小细胞肺癌脑转移临床诊疗指南（2025版）》^[28]^。

**表1:** 推荐强度/证据等级标准

证据等级	标准
A	基于至少一项III期临床研究，或两项及以上I、II期研究
B	基于一项I、II期研究，或观察性、回顾性研究、病例报道
C	基于专家意见

## 2 诊断

### 2.1 临床表现

LM是一种影响从大脑到脊髓的整个神经轴的疾病，早期临床特征往往是非特异性的，可以从无症状到多种神经系统表征^[29]^，多与CSF循环受阻或脑膜炎性刺激有关，2/3的患者有≥2个部位（大脑、后颅窝或脊髓）受累，表现为头痛、喷射性呕吐、复视、下肢无力或尿潴留等^[9]^。因此，当NSCLC患者出现多水平损伤的神经症状，比如复视伴腱反射消失等组合时，应高度警惕LM。此外，由于LM会随着CSF流动而扩散，通常会在CSF流速较慢的后颅窝和马尾处附着^[4]^，从而出现相关神经症状（共济失调、下肢无力、马鞍麻木等）。

### 2.2 影像学表现

#### 2.2.1 磁共振成像（magnetic resonance imaging, MRI）

颅脑和全脊髓增强MRI是诊断LM首选的影像学检查方法。典型影像表现为沿脑沟、脑叶表面、脊髓、颅神经和脊神经分布的线性或结节状异常强化信号。

根据欧洲神经肿瘤学会与欧洲肿瘤内科学会（European Association of Neuro-Oncology and European Society for Medical Oncology, EANO-ESMO）联合指南，基于MRI影像学表现将LM分为4种类型——线性软脑膜病变（A型）、结节状软脑膜病变（B型）、A型与B型两者兼有（C型）、仅有脑积水（D型）或可疑/未见异常影像（D型正常）^[2]^。值得注意的是，增强MRI诊断LM的特异性较高，但敏感性相对有限，部分患者尽管存在LM，常规增强MRI仍可出现假阴性结果，因此MRI阴性并不能完全排除LM的可能^[16]^。为提高检出率，建议采用三维T1加权容积增强序列（three-dimensional T1-weighted contrast-enhanced sequence, 3D T1-CE），以提高对细小或早期病灶的显示^[2,30]^。增强后三维液体衰减反转恢复序列（three-dimensional contrast-enhanced fluid-attenuated inversion recovery sequence, 3D CE-FLAIR）可作为有效补充，能够提高对LM微小病灶的检出率与诊断信心^[31]^。此外，“黑血成像”（black-blood imaging）在检测LM方面显示出较常规增强序列更高的敏感性，但其相较于CE-FLAIR序列并未表现出明显优势^[32]^。

在临床诊断路径上，EANO-ESMO指南进一步将LM划分为I型（CSF细胞学确诊+MRI确诊病例）与II型（临床表现+MRI可疑病例）。研究^[33]^表明，肺癌的LM更倾向于表现为I型，且该型患者预后通常较II型更差。

相较于MRI，计算机断层扫描（computed tomography, CT）对LM的诊断敏感性显著偏低^[34]^，不推荐作为常规评估手段。然而，在急诊情况下或存在MRI禁忌时，增强CT可作为初步筛查工具。

#### 2.2.2 正电子发射断层扫描/计算机断层扫描（positron emission tomography/computed tomography, PET/CT）与PET/MRI

核医学融合型影像设备PET/CT、PET/MRI集成了解剖影像和功能影像的优势，由PET从分子水平上获得脑组织的生理、病理及代谢等信息。显像剂通常使用反映肿瘤细胞葡萄糖代谢活性的^18^F-氟代脱氧葡萄糖（^18^F-flurodeoxyglucose, ^18^F-FDG），已在多种实体瘤LM中显示出诊断价值^[35,36]^。但受PET空间分辨率影响，判定病变位于脑回还是脑膜，主要依靠增强MRI。

LM的^18^F-FDG PET典型表现为沿脑沟分布的线条样、片样或弥漫性代谢增高区，相应CT或MRI示局部脑沟变窄，脑质肿胀^[37]^。对于无法行增强MRI和无法进行CSF穿刺的患者，或增强MRI和CSF阴性但仍怀疑LM的患者，建议行^18^F-FDG PET检查^[38]^。诊断时需注意与脑膜生理性摄取鉴别，后者常见于蛛网膜颗粒区（如矢状窦旁）和颅底脑膜，呈对称性分布^[39,40]^。^18^F-FDG PET/CT半定量分析有助于提高诊断准确性。

氨基酸类示踪剂可更敏感地检测软脑膜病灶，包括^11^C-甲基蛋氨酸（^11^C-methionine,^ 11^CMET）、6-[^18^F]氟-L-多巴（6-[^18^F]-L-fluoro-L-3,4-dihydroxyphenylalanine, ^18^F-FDOPA）以及^18^F-氟乙基酪氨酸（^18^F-fluoroethyltyrosine, ^18^F-FET）等示踪剂。它们通过L型氨基酸转运体（L-type amino transporter, LAT-1）特异性进入肿瘤细胞，而在正常脑组织的摄取较少，能够更清楚地显示肿瘤组织与脑本底分界，提高脑膜病变检出率^[41]^。对于无法行增强MRI和无法进行CSF穿刺的患者，或增强MRI和CSF阴性但仍怀疑LM的患者，可推荐氨基酸类示踪剂PET检查进行补充。

PET/MRI集成了MRI高软组织分辨率、多参数、多序列、低辐射和PET功能代谢的优势，能够同时获得脑结构、功能和分子代谢信息，可以提升肺癌LM检出率，未来应用前景广泛。

### 2.3 CSF细胞学及生化检查

CSF细胞学检查是诊断LM的传统金标准方法，然而，形态学的首次检查敏感性有限，通常为50%-90%^[42]^。有研究^[43,44]^表明CSF首次采样阳性率约50%，重复采样阳性率可增加至75%-85%，但更多次数的采样并不能增加更多阳性率。故如果第一次采样未发现肿瘤细胞，必要时可进行多次采样；采样量应≥5 mL，>10 mL为最优^[2,45]^，标本应及时处理（最好30 min内）^[44,46]^。

CSF细胞学检查常用Cytospin（离心甩片法）和Thinprep（薄层液基细胞学检测法）制片。离心甩片法快速、经济， 适用于常规筛查和初步诊断^[47]^；薄层液基制片自动化程度高，但可能一定程度上影响细胞形态，或存在细胞丢失。在实际工作中建议将两种方法结合使用，或根据临床初步诊断和样本情况灵活选择，以取长补短，达到最佳的诊断效果。对于细胞量大的CSF标本可制作细胞蜡块进行染 色检测，以判定肿瘤细胞及其分型^[48]^。

除细胞学检查外，CSF检查可包括评估压力、颜色、细胞及生化指标。LM常见葡萄糖下降、蛋白升高，肿瘤标志物如癌胚抗原（carcinoembryonic antigen, CEA）、细胞角蛋白19片段抗原（cytokeratin 19 fragment, CYFRA21-1）升高具有诊断价值^[49,50]^。

### 2.4 CSF液体活检及基因检测

CSF液体活检技术主要包括CSF循环肿瘤DNA（circulating tumor DNA, ctDNA）和CSF循环肿瘤细胞（circulating tumor cells, CTCs）的检测，已成为LM诊断和动态监测的重要辅助手段。

对驱动基因阳性NSCLC患者的LM诊断，除了“有没有转移”，还需要知悉的是其基因型。因此，CSF ctDNA是目前除外科活检外获取CSF中肿瘤细胞基因型的成熟方式。CSF ctDNA检测主要通过聚合酶链式反应（polymerase chain reaction, PCR）、下一代测序（next-generation sequencing, NGS）、全基因组测序（whole-genome sequencing, WGS）等技术手段，分析肿瘤细胞释放至CSF的DNA片段，可提供驱动基因突变、拷贝数变异、单核苷酸变异等信息，不仅能显示LM患者与原发肿瘤基因变异一致的分子特征，更能显示与原发肿瘤不同于原发灶的分子特征，且较血浆样本更灵敏^[51]^，丰度更高^[52]^，更能反映出LM患者颅内的分子微环境^[53]^。吴一龙团队^[54]^分析了584例NSCLC伴CNS转移患者的CSF ctDNA，证实CSF ctDNA检测到驱动基因阳性的患者可从相应靶向治疗中改善生存获益（HR=0.78, P=0.003）；而且纵向监测CSF可识别CNS特异性耐药机制，指导第二次匹配靶向治疗进一步改善生存（HR=0.56, P=0.018）。尽管CSF ctDNA在LM的分子诊断与检测中显示出更高的灵敏度和信息量，但是ctDNA的可及性和价格可能限制其成为临床首选诊断方法。

CSF CTC液体活检技术利用特异性抗体捕获（稀有细胞捕获技术）并计数CSF中的肿瘤细胞，可以定量监测LM肿瘤细胞的动态变化^[55]^，更适宜悬浮态的LM的早期诊断和疗效评估。在一项小样本的诊断研究^[56]^中，CSF CTC诊断实体瘤LM的敏感性为93%，特异性为95%，远超MRI和CSF细胞学，并建议至少1个CTC/mL作为LM诊断的最佳阈值；另一项评价CTC计数在人表皮生长因子受体2（human epidermal growth factor receptor 2, HER2）阳性癌症患者中评估肿瘤负担和治疗反应的研究^[57]^发现，75%的患者在CTC中确认HER2表达，并能够提前2-3个月预测影像学变化，而CSF细胞学结果可能仍然为阴性。尽管CTC检测目前受限于技术可及性和大样本临床证据的缺乏，仍需进一步验证，但专家组认为其可作为CSF细胞学阴性但高度怀疑LM患者的辅助诊断手段，具有重要的临床应用前景。

### 2.5 外科活检

在极少数情况下，如果原发癌不明，或者CNS影像学表现不典型，且无法通过CSF细胞学检查确诊LM，可考虑进行柔脑膜或硬膜活检以获取组织进行病理诊断^[58]^。

**共识1：NSCLC LM的诊断应基于临床症状和体征、MRI增强影像和CSF细胞学进行综合诊断，并以细胞学为主要判断依据（A）。**

**共识2：针对增强MRI和CSF细胞学均阴性但临床仍怀疑LM的患者，可推荐氨基酸类示踪剂PET检查进行补充（B）。**

**共识3：NSCLC LM患者CSF细胞学采样和诊断流程应遵循多量采样、及时处理的原则（C）。**

**共识4：CSF ctDNA可以用于明确NSCLC LM患者的驱动基因突变类型和疗效评估（A）。**

**共识5：CSF CTC检测可作为CSF细胞学阴性并怀疑LM患者的辅助诊断（B）。**

## 3 治疗

### 3.1 治疗原则

驱动基因阳性NSCLC患者LM的治疗应基于MDT讨论，并遵循个体化原则。传统上，基于LM的双形态特性，EANO-ESMO将实体瘤LM的治疗依据细胞学和影像学诊断评估做了分层，建议鞘内注射为主导、辅以全身和局部放疗的综合治疗方案^[2]^。但如本共识引言部分所述，对于驱动基因阳性NSCLC，新型靶向药物可以透过BBB进入CSF^[21,22]^，甚至有的靶向药物通过提高剂量或联合抗血管生成治疗可进一步增强疗效，实现显著或完全缓解^[59,60]^。因此，靶向治疗应作为驱动基因阳性NSCLC LM的主要干预手段，局部治疗（鞘内注射化疗和局部放疗）则作为辅助治疗手段（图1）。

**图1:**
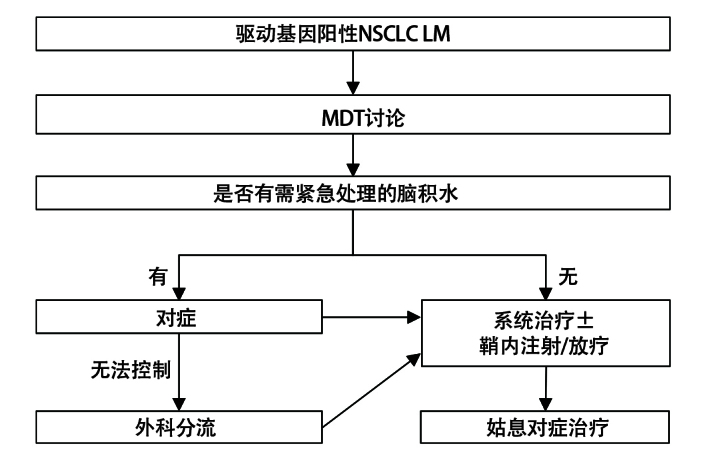
驱动基因阳性NSCLC LM治疗流程图。NSCLC：非小细胞肺癌；MDT：多学科诊疗；LM：脑膜转移。

关于局部治疗的治疗时机目前仍没有定论。有共识^[61]^认为应在全身治疗无效后才考虑鞘注化疗药物，即在酪氨酸激酶抑制剂（tyrosine kinase inhibitors, TKIs）耐药后或LM进展再联合局部治疗。但近年来随着第三代EGFR-TKIs的临床普及，对于一线EGFR-TKIs治疗后出现的LM，可以考虑联合鞘内注射，但这种联合方案主要基于专家经验，而非大规模随机对照试验（具体参见3.1.2节）。鞘内注射药物的选择不应局限于传统化疗药物（甲氨蝶呤、阿糖胞苷等），而应探索更有效的方案。放疗则应避免既往全脑照射，在可接受的生活质量下延长生存期，并预防或延缓神经功能恶化。关于LM疗效的评估，EANO-ESMO认为每6-12周进行一次颅脑-脊髓MRI或CSF细胞学监测^[2]^，但均缺少临床研究的佐证。

另外，相当比例的LM患者伴有脑积水，建议给予积极的对症治疗，如高渗透性药物甘露醇联合激素可短暂缓解脑积水导致的颅高压表现，必要时应及时通过外科手段减轻脑积水，避免出现神经系统急症。

**共识6：NSCLC LM患者的治疗方案应基于充分的MDT讨论制定（A）。**

**共识7：对于EGFR和ALK阳性的、初诊的NSCLC LM，应优选靶向治疗；视临床症状±鞘内注射化疗药物（A）。**

### 3.2 靶向治疗

NSCLC靶向药物在CSF中的有效渗透是治疗LM的关键，受血CSF屏障的双向调节机制影响，药物进入CSF的过程受到流入机制（如被动扩散、胞吞、受体/载体介导转运等）和外排机制（如P-糖蛋白泵出）共同影响^[62]^。部分靶向药物不是外排蛋白底物，如佐利替尼、阿来替尼^[63]^、洛拉替尼^[64]^；另外部分靶向药物虽然是外排蛋白的底物，但其高渗透性可克服外排作用，亦能在CSF中达到治疗浓度，如奥希替尼^[65]^。但总体而言，第三代EGFR-TKIs和第二、三代ALK-TKIs均有较好的CSF渗透率和显著的治疗效果，安全性亦优于传统放疗和鞘内化疗。循证方面，因LM更多地发生于EGFR突变和ALK融合的NSCLC患者，因此前瞻性LM的靶向治疗证据目前主要集中在EGFR和ALK两个靶点，对其他的驱动基因靶向治疗也有一定借鉴意义。

#### 3.2.1 EGFR突变

##### 3.2.1.1 初治EGFR突变患者合并LM

EGFR-TKIs是目前EGFR突变NSCLC的标准首选治疗。由于新型EGFR-TKIs CSF渗透率显著提升，LM患者的中位OS普遍提升到1年左右（表2^[59,60,66-71]^）。虽然第一、二代EGFR-TKIs对NSCLC LM人群也有颅内反应，但OS并没有得到显著的提升，中位OS维持在4个月以下^[67]^，但迭代到第三代EGFR-TKIs后，对CNS的渗透率明显增强，如奥希替尼血浆和CSF的药物浓度比为16%^[60]^，高于阿法替尼的2.5%^[72]^。

**表2:** EGFR-TKIs治疗NSCLC伴LM的临床研究汇总

文献	研究类型	患者类型	治疗组设计	n	中位OS	其他结果
Tamiya A, 2017^[72]^	前瞻性研究^a^	初治	阿法替尼40 mg/d	11	3.8（95%CI: 1.1-13.1）个月	中位PFS：2.0（95%CI: 0.6-5.8）个月；ORR：27.3%
Nosaki K, 2020^[67]^	II期	初治	厄洛替尼150 mg/d	21	3.4个月	CSF细胞学清除率：30.0%（95%CI: 11.9%-54.3%）
Lu ZQ, 2021^[59]^	II期	初治	奥希替尼80 mg/d+贝伐珠单抗7.5 mg/kg，每3周1次	14	12.6（95%CI: 9.8-21.2）个月	LM ORR：50%；中位LM PFS：9.3（95%CI: 8.2-10.4）个月
Jackman DM, 2015^[68]^	I期	经治	高剂量吉非替尼750-1000 mg/d（2周）+维持剂量吉非替尼500 mg/d（2周）	7	3.5（1.6-5.1） 个月	中位CNS PFS：2.3（1.6-4.0）个月
Nanjo S, 2018^[69]^	前瞻性研究^a^	经治	奥希替尼80 mg/d	13	NR	中位PFS：7.2（95%CI: 4.0-无法确定）个月
Yang JCH, 2020^[60]^	I期	经治	奥希替尼160 mg/d	41	11（95%CI: 8.0-18.0）个月	LM ORR：62%（95%CI: 45%-78%）；LM缓解持续时间：15.2（95%CI: 7.5-17.5）个月；中位PFS：8.6（95%CI: 5.4-13.7）个月
Park S, 2020^[70]^	II期	经治	奥希替尼160 mg/d	40	13.3（95%CI: 9.1-NR）个月	中位PFS：8.0（95%CI: 7.2-NR）个月；颅内疾病控制率：92.5%
Qi R, 2023^[71]^	前瞻性研究^a^	经治	伏美替尼160 mg/d± 抗血管生成药物	16	NA	中位颅内PFS：4.3（95%CI: 2.1-6.5）个月

^a^：分期未注明。EGFR：表皮生长因子受体；TKIs：酪氨酸激酶抑制剂；NR：未达到；NA：未报道；PFS：无进展生存期；OS：总生存期；ORR：客观缓解率；CSF：脑脊液；CNS：中枢神经系统。

EGFR-TKIs联合抗血管生成药物（如贝伐珠单抗）被视为EGFR突变NSCLC LM的改进治疗策略，其理论基础是血管内皮生长因子（vascular endothelial growth factor, VEGF）和EGFR共享部分下游通路^[73]^。初步研究^[59]^显示，奥希替尼联合贝伐珠单抗治疗LM的颅内客观缓解率（objective response rate, ORR）为50%，中位PFS达9.3个月，与提高第三代EGFR-TKIs剂量治疗LM的研究结果相似，但联合治疗对OS的提升作用有限，且目前证据主要来源于小样本回顾性研究，尚需大规模随机对照试验进一步验证。另外值得注意的是，对于合并脑水肿或放射性神经损伤的患者，联合抗血管生成药物可能带来额外获益^[74]^，因此在临床实践中应个体化权衡风险与获益。

初始诊断EGFR阳性NSCLC即伴有LM的患者并不多见，建议首选第三代EGFR-TKIs，通常有较好的疗效，在治疗前期可不联合鞘注化疗。

##### 3.2.1.2 EGFR-TKIs靶向治疗期间出现LM

对于在EGFR-TKIs治疗期间出现的LM，临床上通常有3种情况（图2）：（1）多数经第一或二代EGFR-TKIs治疗后出现LM；（2）经奥希替尼等第三代EGFR-TKIs一线标准治疗，治疗期间仅出现LM进展；（3）第三代EGFR-TKIs治疗后出现了包含LM在内的多部位进展/转移。

**图2:**
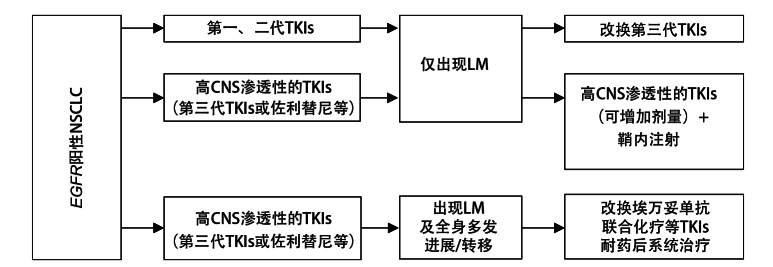
EGFR阳性NSCLC接受EGFR-TKIs治疗过程中出现的LM治疗流程图。

针对第一种情况，改用具有高CNS渗透性的TKIs（如新型TKIs佐利替尼、第三代TKIs奥希替尼等）进行单药维持治疗可能是合理的。III期EVEREST研究LM合并脑实质转移的EGFR阳性NSCLC患者亚组中，佐利替尼相较第一代EGFR-TKIs可显著降低颅内PFS风险达60%（HR=0.395）^[75]^。因此，高CNS渗透性的TKIs可带来更持久且稳健的颅内病灶控制，此时无需立即联合局部治疗LM。

针对第二种情况，有研究^[60]^探索了EGFR-TKIs剂量的调整和优化。BLOOM研究评估了双倍剂量奥希替尼（160 mg qd）在已接受过EGFR-TKIs治疗的NSCLC LM患者中的效果；结果显示28%的患者肿瘤细胞在CSF中被清除，57%的患者神经症状得到改善，持续反应时间（duration of response, DoR）为15.2个月，此外中位PFS为8.6个月，中位OS为11.0个月。另一项前瞻性真实世界研究评估了高剂量伏美替尼（240 mg qd）在EGFR突变NSCLC LM患者中的疗效与安全性（n=48），结果显示临床反应率为75%，ORR和疾病控制率（disease control rate, DCR）分别为50%和92.1%^[21]^。此外，也有佐利替尼与伏美替尼作为后线的病例报道，显示出快速症状缓解和中枢病灶控制的潜在效果^[76]^。值得注意的是，提高第三代EGFR-TKIs剂量治疗LM只有部分II期研究结果，仍缺乏随机对照的证据，且所带来的CNS控制时间相对有限；考虑到EGFR-TKIs耐药后LM的侵袭性，联合鞘内化疗可能是必要的强化治疗手段，尽管这一联合方案主要基于病例报告和专家经验。

针对第三种情况，对LM的治疗策略可以更换靶向治疗方案（如埃万妥单抗±化疗）并联合鞘内注射治疗；针对全身进展可结合中国临床肿瘤学会等肺癌指南中靶向药耐药后广泛进展的系统治疗策略。Yu等^[77]^在2024年美国临床肿瘤学会年会上汇报了一项纳入21例LM、奥希替尼经治患者的单臂II期研究，结果发现，改用埃万妥单抗联合拉泽替尼后，治疗反应率为33%，中位PFS为8.3个月，中位OS为14.4个月；提示在第三代TKIs治疗进展后的LM患者可以改用埃万妥单抗改善预后。也有报道^[78]^称埃万妥单抗联合化疗相比单纯化疗显著提升TKIs耐药后CNS转移患者的PFS。

#### 3.2.2 ALK重排

ALK重排NSCLC患者中，约5%会发展为LM^[79]^。ALK抑制剂至今已历经三代，第二、三代ALK抑制剂对颅脑的渗透率良好，特别是洛拉替尼受外排蛋白影响较小^[80]^，CSF浓度接近血浆浓度的73%^[81]^。阿来替尼和洛拉替尼对脑实质转移疗效明确并有长达5年随访的OS证据，但针对LM人群却鲜有前瞻性临床研究。

多项回顾性研究和真实世界数据表明，ALK-TKIs对LM具有局部缓解作用。在洛拉替尼的真实世界研究中（n=11），既往TKIs治疗进展至LM的ALK阳性患者颅内ORR达45%，疾病控制率为91%，其中2例达到颅内完全缓解^[82]^。阿来替尼在多项研究中显示出对LM的挽救疗效。一项研究^[83]^纳入4例克唑替尼/塞瑞替尼治疗后进展至LM的ALK阳性NSCLC患者，其中3例患者的神经系统症状（头痛、复视、言语与认知障碍、肢体无力）及影像学表现均明显改善，脑膜强化近乎消失。在一项中国多中心回顾性研究^[84]^中，8例合并LM的ALK阳性NSCLC患者接受阿来替尼治疗后，中枢相关症状显著缓解，需要甘露醇或皮质类固醇治疗的患者数量显著降低，提示阿来替尼可延迟或减少LM患者的局部治疗需求。此外，个案报告^[85,86]^也支持ALK-TKIs治疗可改善LM负荷和神经系统症状。

**共识8：EGFR突变阳性NSCLC患者合并LM，一线治疗优选高CNS渗透性的TKIs，如奥希替尼（A）、伏美替尼等（A），必要时也可考虑佐利替尼（A）。**

**共识9：对于正在使用EGFR-TKIs靶向治疗的NSCLC患者，如全身症状控制良好但仅新发LM进展时，可以考虑增加原靶向药物的剂量（C）。**

**共识10：对ALK阳性的NSCLC LM，应优选洛拉替尼（A）或阿来替尼（B）。**

### 3.3 鞘内注射

鞘内注射的“鞘”指的是硬膜和蛛网膜，通过鞘内注射，药物可以直接注入到蛛网膜下腔，使药物能直接与肿瘤细胞接触并发挥药效。鞘内注射起源于19世纪末出现的脊神经麻醉^[87]^，直到19世纪70年代才开始尝试鞘内注射化疗，但效果并不理想，并经历了近30年的疗效平台期^[3]^。原因在于，传统化疗药物（如甲氨蝶呤）对结节型的渗透深度有限，仅数毫米，且LM患者常伴CSF动力学障碍（如脑积水），增加了毒性风险，应用受限^[88]^。近十年来，培美曲塞鞘内注射显著改善疗效，其渗透深度更优，对悬浮态和结节型LM均有效。此外，Ommaya储液囊脑室内给药系统的应用，能够将药物直接注射至脑室，显著提升药物在脑室内的分布均匀性，有效降低并发症风险，从而在疗效和操作便捷性方面优化了鞘内注射治疗（详见3.5.2节）。

对于驱动基因阳性的NSCLC LM患者，专家组认为，应该对患者的LM时机进行研判，如果患者携带EGFR敏感突变或ALK重排，在使用第一、二代TKIs的过程中出现LM，尤其是当EGFR-TKIs耐药后检出T790M突变，鉴于靶向治疗在CNS的良好疗效，可优先采用第三代EGFR-TKIs靶向治疗，鞘内注射化疗可择机而行^[2]^。如果患者是在使用第三代EGFR-TKIs或第二代ALK-TKIs的过程中出现LM，如果不伴有颅外病灶的进展，建议使用高剂量第三代EGFR-TKIs或第三代ALK-TKIs洛拉替尼，可联合鞘注化疗（建议培美曲塞）。如果合并颅外病灶的进展，建议更改系统性治疗方案，酌情联合鞘内注射化疗。需要强调的是，目前的治疗方案选择、最佳应用时机、治疗周期、鞘注治疗停药时机均缺乏高质量循证证据，临床决策应综合患者病情及MDT，实施个体化治疗。

目前已有多项针对培美曲塞的实体瘤鞘注化疗研究报道，疗效明确且耐受性可控。一项开放单臂、I/II期临床试验^[89]^探讨了EGFR突变NSCLC LM靶向治疗失败后再使用鞘内注射的可行性：研究者使用培美曲塞和地塞米松的联合方案，其中培美曲塞使用了加速剂量递增给药方式，从15 mg逐步增加到80 mg，结果培美曲塞鞘内化疗显示出84.6%的临床反应率，显著高于传统的鞘内甲氨蝶呤、阿糖胞苷和噻替哌注射（53%-79%）；此外，在TKIs耐药后，培美曲塞方案仍能达到9个月的中位OS获益。另一项更大样本量（n=132）的TKIs耐药后鞘内治疗研究^[25]^也得出了相似的结论，但鞘内注射培美曲塞的策略有所差异，即第1天和第5天给予各50 mg培美曲塞作为诱导治疗，随后每3周1次，共4个周期，再每月1次，直至疾病进展或不耐受；结果显示中位OS为12个月，80.3%的患者显示出治疗反应，14.4%的患者疾病稳定，5.3%患者疾病进展；最常见的不良反应为骨髓抑制（31.8%），大多数不良反应为1或2级。因此，对于驱动基因阳性NSCLC LM靶向治疗失败的患者，应优选培美曲塞进行鞘内注射治疗。

**共识11：NSCLC LM患者鞘内注射治疗用药应优选培美曲塞。**

### 3.4 放疗

目前尚无针对LM的高质量I类放疗临床研究证据，因此放疗策略主要基于现有文献报道、专家共识及临床经验^[44,90]^，推荐患者参与临床研究。

#### 3.4.1 放疗时机

对于CSF细胞学阳性但影像学尚无明显表现的患者，可考虑延后放疗，此阶段应以系统治疗为主。对于影像学阳性但无明显临床症状的患者，建议行MDT讨论放疗时机。而对于影像学阳性且存在临床症状的患者，则建议积极进行脑部放疗，以稳定或改善神经系统症状。放疗方式的选择需结合病灶范围、临床症状以及患者整体状态，进行个体化评估。

#### 3.4.2 放疗方式选择

对于局限性（结节性）LM患者（伴或不伴脑实质转移），推荐行累及野立体定向放射外科（stereotactic radiosurgery, SRS）/立体定向放疗（stereotactic radiotherapy, SRT）。对于EGFR突变NSCLC LM患者，全脑放疗（whole brain radiotherapy, WBRT）并未显著改善总体生存，因此可优先考虑SRS/SRT的局部精准放疗方案^[91,92]^。具体放疗分割方案可参考《中国驱动基因阳性非小细胞肺癌脑转移临床诊疗指南（2025版）》中的放疗部分^[28]^。

对于广泛性LM患者，可考虑WBRT。而全脑全脊髓放疗（craniospinal irradiation, CSI）需谨慎使用，仅在特定患者且可耐受的情况下考虑^[93]^。

质子CSI显示出潜在优势。文献^[94]^报道，与传统光子CSI相比，质子CSI可延长CNS转移患者的PFS及OS，且毒性差异不明显。然而，由于质子放疗设备在国内仅少数机构可用，目前尚不作为一类推荐。

**共识12：放疗是驱动基因阳性NSCLC LM患者的辅助或缓解性干预措施，应根据病灶范围、临床症状及患者体能综合评估（A）。**

**共识13：局限性病灶优先精准放疗（SRS/SRT），可改善神经症状且降低毒性（C）。广泛病灶或症状明显者，WBRT或CSI可作为选择，但需严格评估毒性与获益（C）。**

### 3.5 外科治疗

由于缺乏针对NSCLC LM神经外科干预的随机对照试验研究并考虑到LM的弥漫性特点，使得手术切除在NSCLC LM中的作用有限，目前主要用于罕见情况下的诊断性活检或症状性局灶的减瘤^[58,95,96]^。因此，神经外科干预通常不以治愈为目的，而是旨在对症治疗或明确诊断、减少肿瘤负荷，为综合治疗争取时间：如CSF分流术处理脑积水、放置Ommaya储液囊进行鞘内化疗等。这与针对可切除原发性或孤立性转移性脑肿瘤的神经外科手术有着本质区别。因为传统的神经外科的“完全切除、长期生存”等成功标准通常不适用于LM。

#### 3.5.1 脑积水的外科处理

1%-5%的LM患者会发生脑积水^[97]^。LM所致脑积水是一种亚急性病变，症状通常在数周而不是数日内发展^[98]^。LM导致的脑积水大多为交通性，可能是转移瘤细胞阻碍了蛛网膜对CSF的重吸收^[59]^。特别是对于恶性颅高压、药物难以缓解的脑积水应采用外科手术治疗缓解症状^[58,99]^。针对LM相关脑积水，CSF分流术（ventriculoperitoneal shunts, VPS）是外科治疗的首选方式^[100]^。尽管VPS对于症状的改善和生活质量的提升是明确的^[101]^，但分流手术对改善生存的作用仍不清楚，一项纳入12项研究共503例LM患者的系统评价发现，CSF分流术后的中位生存期仅为3.1个月^[102]^，低于非手术治疗所达到的生存时间^[72,95,103]^，这可能与LM合并脑积水的患者往往处于疾病的终末阶段，体能状态及疾病控制状况往往较差有关^[104]^。VPS的具体操作和方式选择可参考相关共识^[105]^。

VPS理论上有腹膜播散风险，虽然实际的发生率并不高，但仍需警惕。有系统回顾^[102]^发现，在420例行VPS的LM相关脑积水患者中，仅有1例在全身进展时出现了腹膜转移。另一项回顾性研究^[106]^中腹膜播散转移率仅为3.4%（2/58）。VPS并发症还包括感染、阻塞等，发生率在0%-21.1%，需密切随访^[102]^。

腰大池腹腔分流（lumboperitoneal shunt, LPS）可作为交通性脑积水且全麻耐受差患者的替代治疗，疗效与VPS相当^[100]^。部分学者^[100,104,107]^认为LPS手术操作较简单，损伤较小，感染发生率较低，可能更适合部分LM患者。严重的交通性脑积水也可以行紧急腰穿释放脑积液缓解。此外，LM合并脑积水的手术治疗应结合手术机器人、导航等新技术，优化穿刺与分流流程，减少并发症，联合MDT，在提高患者生存质量的同时，努力延长患者的生存时间。

#### 3.5.2 Ommaya囊的置入

既往的鞘内注射经腰穿给药（腰椎内途径），频繁腰穿会引发患者的焦虑不适、穿刺后并发症风险（硬膜穿刺后头痛、腰痛等），也不能保证药物在CSF中的均匀分布。通过埋于头皮下的储液囊（Ommaya囊）、经引流管直接进入脑室，与腰椎内途径相比，更有利于药物在脑室内及鞘内的扩散^[108]^，可以较方便地抽取CSF评估治疗反应或经Ommaya囊注射药物^[109]^。一项多中心随机对照试验^[110]^比较了脑室内与腰椎内对LM鞘内注射疗效的影响，结果发现对于接受甲氨蝶呤的患者，脑室内给药组的PFS显著优于腰椎内给药组。另一项纳入40例LM患者的比较研究^[109]^也进一步证实，经Ommaya囊化疗患者的OS显著优于腰椎内途径（9.2 vs 4个月）。

在CNS肿瘤指南中，Ommaya囊常被作为强烈推荐，主要适用于需长期或频繁CSF给药或抽取、因解剖或技术原因难以进行腰椎穿刺，或正在接受抗凝/抗血小板治疗的患者。手术禁忌包括梗阻性脑积水、CSF呈胶冻状、预计生存期较短、药物治疗不敏感（原发或继发耐药）、无法正确操作装置，以及常规手术禁忌（如全身状况差、活动性感染或正在接受抗凝及抗血小板治疗）等。尽管Ommaya囊与VPS或LPS的联合手术在理论上可以同时解决脑积水（颅内高压）和脑室内给药的需求，但在实践中，这种组合带来了显著的技术挑战和并发症风险，尤其是感染^[108]^。因此，该手术方案的决策应极为审慎，通常只适用于没有更好替代方案且预期获益大于潜在风险的复杂病例。同时，Ommaya囊植入手术的诊断与治疗价值也应结合“疾病诊断相关分组（diagnosis-related groups, DRG）”支付政策讨论。

**共识14：手术切除在已确诊的LM中作用非常有限，一般不推荐。脑膜活检主要用于罕见情况下的完善诊断。如瘤体巨大、局限且症状显著，可行减瘤手术，为后续综合治疗争取时间（A）。**

**共识15：对于需要鞘内注射的患者，推荐Ommaya囊植入（A）。**

**共识16：对于颅内压增高症状明显的脑积水患者，应优先考虑脑室腹腔分流或Ommaya囊植入（A）。腰大池腹腔分流可作为交通性脑积水且全麻耐受差患者的治疗替代选择（B）。**

### 3.6 对症治疗 支持治疗在NSCLC

LM中以缓解症状、改善生活质量为核心^[2]^。LM通常伴随着严重的症状，如疼痛、癫痫、认知障碍等，这些症状显著影响患者的生活质量。虽然LM的预后通常较差，但有效的支持治疗有助于延长患者的生存期^[111]^。对于有脑水肿伴颅内压升高症状的患者，可短期使用糖皮质激素（如地塞米松8-16 mg/d起），以及甘露醇脱水剂等，症状缓解后应尽早减量，以减少不良反应^[112]^。癫痫管理避免一级预防^[100]^，首次发作后可选择与抗肿瘤药物相互作用少的抗癫痫药物，如左乙拉西坦^[113]^。认知障碍可使用NMDA受体拮抗剂（如美金刚）或非药物干预（如心理支持）缓解^[114,115]^。其他与脑转移相似症状可参考《中国驱动基因阳性非小细胞肺癌脑转移临床诊疗指南（2025版）》^[28]^。

## 4 免责声明

本专家共识旨在为相关领域的肿瘤专科医师提供临床决策参考，仅适用于驱动基因阳性的NSCLC患者所发生的LM。文中观点与建议系基于既有证据和专家经验形成，不应被视为绝对正确的结论，亦不构成固定不变的标准治疗方案或临床实践规范，更不能替代临床专科医生在具体情境中的独立判断。本共识所依据的研究证据主要来源于截至2025年10月前收录于PubMed及相关书籍的文献，因而可能无法完全反映其后出现的最新循证进展。

**指南编写组成员**

**组长：**支修益（首都医科大学宣武医院），王洁（中国医学科学院肿瘤医院）

**执笔：**赵军（北京大学肿瘤医院暨北京市肿瘤防治研究所，恶性肿瘤发病机制及转化研究教育部重点实验室），李晓燕（首都医科大学附属北京天坛医院）

**成员（按姓氏拼音排序）：**曹宝山（北京大学第三医院），蔡修宇（中山大学肿瘤防治中心），蔡洪庆（中国医学科学院肿瘤医院），陈麦林（北京大学肿瘤医院），陈绪珠（首都医科大学附属北京积水潭医院），褚倩（华中科技大学同济医学院附属同济医院），常笑（北京大学肿瘤医院），戴朝霞（大连医科大学附属第二医院），段建春（中国医学科学院肿瘤医院），冯晨璐（首都医科大学附属北京天坛医院），胡牧（首都医科大学附属北京友谊医院），胡毅（中国人民解放军总医院），黄鼎智（天津医科大学肿瘤医院），金波（中国医科大学附属第一医院），李囡（北京大学肿瘤医院），李子明（上海市胸科医院），刘玉良（北京大学肿瘤医院），农靖颖（首都医科大学宣武医院），商琰红（河北大学附属医院），石安辉（北京大学肿瘤医院暨北京市肿瘤防治研究所），宋天彬（首都医科大学宣武医院），苏春霞（上海市肺科医院），孙时斌（首都医科大学附属北京天坛医院），汤传昊（北京大学国际医院），汪进良（中国人民解放军总医院），王玉栋（河北医科大学第四医院），王志杰（中国医学科学院肿瘤医院），王玺（北京大学肿瘤医院），吴芳（中南大学湘雅二医院），邬麟（湖南省肿瘤医院），万经海（中国医学科学院肿瘤医院），徐蔚然（首都医科大学附属北京天坛医院），杨明（中国医学科学院肿瘤医院），杨波（中国人民解放军总医院派驻第八医学中心），杨晨（空军军医大学唐都医院），杨萌（中日友好医院），杨雪（北京大学肿瘤医院），杨宇（哈尔滨医科大学附属第二医院），燕翔（北京大学人民医院），姚煜（西安交通大学第一附属医院），易福梅（北京大学第三医院），张明山（首都医科大学三博脑科医院），张弨（首都医科大学附属北京天坛医院），张燕（河北医科大学附属人民医院），赵晖（首都医科大学附属北京天坛医院），赵博（北京大学肿瘤医院），周进（四川省肿瘤医院），周清（广东省人民医院），周永（新疆医科大学附属肿瘤医院），庄洪卿（北京大学第三医院），卓明磊（北京大学肿瘤医院），朱翔（北京大学医学部/北京大学第三医院）

**特邀顾问：**杨学军（清华大学北京清华长庚医院），焦顺昌（中国人民解放军总医院肿瘤医学部派驻第一医学中心），李琳（北京医院）
